# LHP1 Regulates H3K27me3 Spreading and Shapes the Three-Dimensional Conformation of the Arabidopsis Genome

**DOI:** 10.1371/journal.pone.0158936

**Published:** 2016-07-13

**Authors:** Alaguraj Veluchamy, Teddy Jégu, Federico Ariel, David Latrasse, Kiruthiga Gayathri Mariappan, Soon-Kap Kim, Martin Crespi, Heribert Hirt, Catherine Bergounioux, Cécile Raynaud, Moussa Benhamed

**Affiliations:** 1 Division of Biological and Environmental Sciences and Engineering, King Abdullah University of Science and Technology, Thuwal, 23955-6900, Kingdom of Saudi Arabia; 2 Institute of Plant Sciences Paris-Saclay (IPS2), CNRS, INRA, University Paris-Sud, University of Evry, University Paris-Diderot, Sorbonne Paris-Cite, University of Paris-Saclay, Batiment 630, 91405, Orsay, France; Ecole Normale Superieure, FRANCE

## Abstract

Precise expression patterns of genes in time and space are essential for proper development of multicellular organisms. Dynamic chromatin conformation and spatial organization of the genome constitute a major step in this regulation to modulate developmental outputs. Polycomb repressive complexes (PRCs) mediate stable or flexible gene repression in response to internal and environmental cues. In *Arabidopsis thaliana*, LHP1 co-localizes with H3K27me3 epigenetic marks throughout the genome and interacts with PRC1 and PRC2 members as well as with a long noncoding RNA. Here, we show that LHP1 is responsible for the spreading of H3K27me3 towards the 3’ end of the gene body. We also identified a subset of LHP1-activated genes and demonstrated that LHP1 shapes local chromatin topology in order to control transcriptional co-regulation. Our work reveals a general role of LHP1 from local to higher conformation levels of chromatin configuration to determine its accessibility to define gene expression patterns.

## Introduction

During development of multicellular organisms, early embryonic cells must adopt particular gene expression patterns to differentiate into a variety of functionally specialized cells in tissues and organs [1]. Eukaryotic DNA is wrapped in the nucleus around histone octamers into nucleosomes conforming chromatin structure in association to other proteins and RNA. Covalent modifications of DNA and histones may recruit protein complexes to remodel chromatin, altering its compaction and shaping the three-dimensional topology of the genome. Chromatin structure and genome topology are sensitive to regulatory cues and determine the accessibility of DNA to transcription factors in order to dynamically modulate gene transcription and other genome functions [2].

Polycomb group (PcG) proteins participate in one of the earliest epigenetic regulatory mechanisms identified, the repression of *Hox* genes in *Drosophila melanogaster* [3]. PcGs form multi-subunit Polycomb Repressive Complexes (PRCs), first described in *Drosophila* as PRC1 and PRC2. Traditionally, PRC2 and PRC1 complexes were thought to function sequentially on their target genes, PRC2 being recruited first to promote H3K27me3 deposition and thereby forming a docking site for PRC1 that would subsequently induce further histone modifications and repress gene expression. However, a number of reports have challenged this model and relationships between PRC1 and PRC2 components are probably far more complex than initially anticipated. Indeed, some PRC1 complexes can be recruited independently of H3K27me3 deposition and there is evidence for PRC1-dependent PRC2 recruitment on some loci, suggesting that PRC1 and PRC2 can be recruited independently on chromatin and generate binding sites for each other [4–6]. Since their identification, PcG proteins have been found to exert key roles in epigenetic memory in various biological processes, such as X-chromosome inactivation [7], prevention of senescence [8–10] and stem cell maintenance [11], as well as imprinting in plants [12] and mammals [13]. In addition to their role in the establishment of stable gene expression patterns during development, we now know that PRC-mediated epigenetic modifications also participate in the dynamic response to developmental and environmental cues [14].

In *Arabidopsis*, three distinct PRC2s can be found, based on the characteristic presence of one of the three animal Suppressor of zeste 12 (Su(z)12) homologs: Embryonic Flower 2 (EMF2, [15]), VeRNalisation 2 (VRN2, [16]), or Fertilization Independent Seed 2 (FIS2, [17]). *Arabidopsis* has three Histone Methyltransferases: Curly LeaF (CLF, [18]), SWiNger (SWN, [17]), and MEDEA (MEA/FIS1, [19]). CLF and SWN play partly redundant roles within the EMF and VRN complexes [17, 20], whereas MEA is part of the FIS complex [21]. As in animals, plant PRC2 can introduce the repressive mark H3K27me3 [22–27], which is mostly lost in the *Arabidopsis clf/swn* double mutants [28].

LIKE HETEROCHROMATIN PROTEIN 1 (LHP1 also known as TERMINAL FLOWER 2, TFL2) was identified as the homolog of animal HP1 based on the presence of a chromodomain and a chromoshadow domain [29]. However, HP1 is capable to bind to heterochromatic regions of the genome, whereas LHP1 localizes to euchromatin, controlling actively transcribed genes [30, 31], and is now recognized as a plant specific PcG member [2, 32]. LHP1 occupancy co-localizes with H3K27me3 across the *Arabidopsis* genome [33, 34], and common genes are up regulated in the *lhp1* and *clf* mutants [25, 30]. LHP1 can directly interact with members of both the PRC1 and the PRC2 complexes [35–38] suggesting that LHP1 may act as a bridge between them [39, 40]. In the context of DNA replication, LHP1 binds to H3K27me3 [32] and interacts with PRC2 [39] and with the DNA polymerase ε catalytic subunit EARLYINSHORTDAYS7 (ESD7; [41]). In addition, LHP1-mediated repression requires the DNA polymerase α catalytic subunit INCURVATA 2 (ICU2; [42, 43]). LHP1 thus likely mediates the reestablishment of H3K27me3 in dividing cells [39, 40], by interacting with specific partners. More recently, it was shown that LHP1 interacts with the *APOLO* long noncoding RNA during the dynamic regulation of chromatin three-dimensional conformation in response to auxin [14]. However, the role of LHP1 in the control of genome topology remains unclear. In this work, we show that LHP1 controls the spreading of the H3K27me3 repressive mark, impacting the transcription of euchromatic genes using genome-wide approaches. Moreover, Hi-C analyses revealed that global genome topology is altered in the *lhp1* background and a high correlation of gene transcription between loci positioned at both ends of a same chromatin loop was established. This work challenges the current model of LHP1 in chromatin remodeling and integrates the action of PRC complexes in all levels of genome organization.

## Results

### Genome-wide LHP1 occupancy correlates positively with H3K27me3 and negatively with RNA Pol II

**Fig 1:**
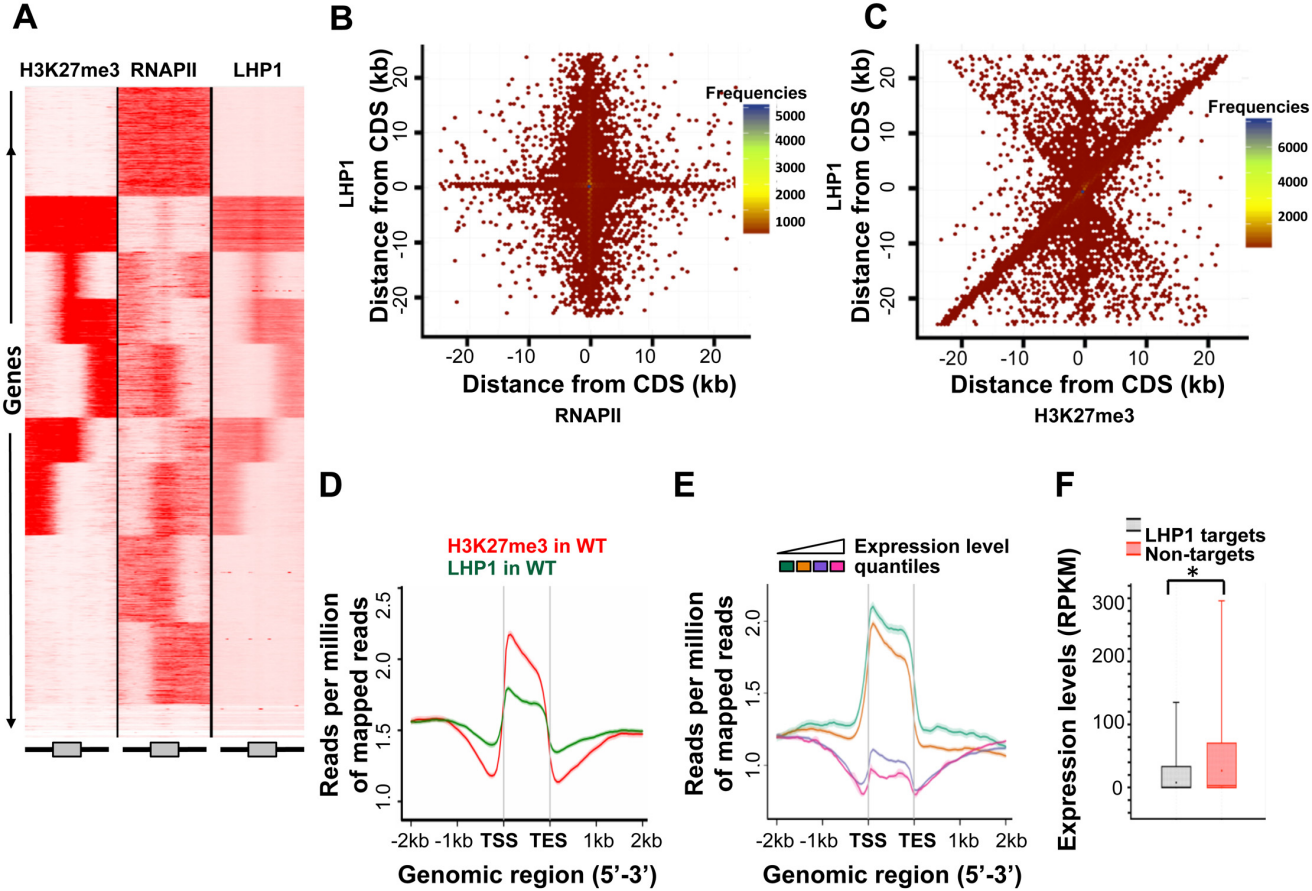
LHP1 occupancy across the *Arabidopsis* genome. **(A)** Correlation between the genome-wide distribution of H3K27me3, RNA Pol II and LHP1. Tag density distribution of LHP1 in WT along with H3K27me3 and RNA Pol II across the genes with 2kb flank, as indicated in the scheme below de graph, gene in grey, flanking regions as black lines. Regions are clustered using K-means linear clustering according to tag density profiles. Density profiles were generated using density array method in seqMINER. Darker red indicates higher density of reads. On the Y-axis there is the list of all genes in TAIR10 annotation. Ten clusters are shown here, revealing the co-occupancy of LHP1 and H3K27me3 and a negative relationship between LHP1 and RNA Pol II. **(B)** Density plot showing overlap of LHP1 and RNA Pol II using Hexagonal binning routine. As large number of data points may overlap, Hexagonal binning gives additional dimension of differentiation of overlapping points based on count. Each point represents the distance of midpoint of peak to nearest gene. On the Y-axis is location of midpoint of LHP1 peak in comparison to gene position; X-axis is location of midpoint of RNA Pol II in comparison to mid-point of gene. This reveals peaks of RNA Pol II and LHP1 do not co-occur physically in the genome. **(C)** Hexagonal binning plot showing the association of LHP1 peak region to that of H3K27me3. Each point represents the distance of midpoint of peak to nearest gene. Most of the peaks overlap on the coding region. Large number of points occurs along the positive correlation line, showing the co-occurrence pattern of LHP1 and H3K27me3. (**D)** Average ChIP-seq enrichment profiles plot of H3K27me3 and LHP1 in WT, stratified by gene length. Normalization of coverage using spline algorithm was performed over the genes and flanking 2 kb region. (**E)** Average enrichment profile of LHP1 is correlated with gene expression variations. Gene expression is categorized from low (first quantile) to high expression (fourth quantile). Mean-normalized ChIP-Seq densities of equal bins along the gene and 2-kb region flanking the TSS or the TES were plotted. Highly expressed genes show lower enrichment for binding of LHP1. (**F)** Boxplot showing the comparison of expression levels in RPKM of LHP1-targeted genes and non-targeted genes in WT. LHP1 targeted genes show lower expression levels. (*) represents Mann–Whitney–Wilcoxon test between LHP1 target and non-target with a p-value < 2.2e-16.

Previous studies based on ChiP-chip [33] and tilling arrays [34] showed that LHP1 occupancy correlates with the H3K27me3 repressive mark predominantly in euchromatic regions of the *Arabidopsis* genome. Hence, we analyzed LHP1 binding sites via ChIP-Seq, as well as the distribution of H3K27me3 and Pol II to assess occupancy correlations and expand previous results to the complete genome. We identified 13,890 peaks for LHP1, corresponding predominantly to genic regions. Clustering the normalized tag-density of LHP1-targeted genes showed a specific enrichment profile highly correlated with H3K27me3 and negatively correlated with RNA Pol II (Fig 1A). According to this set of clusters, read map density is high in the gene-body and spreads along the 2kb flanking region. Comparison of the enrichment profiles of LHP1 and the repressive mark H3K27me3 showed co-occupancy with a specific common pattern of enrichment over the genes. Our analysis identified 8,882 genes recognized by LHP1, most of which not correlated with RNA Pol II occupancy (Fig 1B). Notably, in contrast to RNA Pol II, LHP1 co-localizes with H3K27me3 in a large number of the target genes (Fig 1C). When we assessed the distribution of LHP1 along the genes and their flanking regions, we observed a significant enrichment across the gene body, along with H3K27me3 (Fig 1D). Furthermore, both for LHP1 and H3K27me3 signals were stronger at the TSS and gradually diminish towards the TES.

In order to correlate LHP1 binding with the transcriptional activity of its target genes, we assessed their behavior from Wild Type (Col0, WT) RNA-seq dataset. Stratifying the LHP1 targeted genes as quantiles based on their expression level (higher expression as higher quantile), showed that LHP1 occupancy increases inversely with expression level (Fig 1E). On the whole, the repressive effect of LHP1 in terms of transcription can be appreciated when the distribution of the expression levels of LHP1-marked genes is compared (Mann–Whitney–Wilcoxon test p-value < 2.2e-16) with the one of LHP1-unmarked genes (Fig 1F).

### Dual role of LHP1 on transcriptional regulation is context-dependent

**Fig 2:**
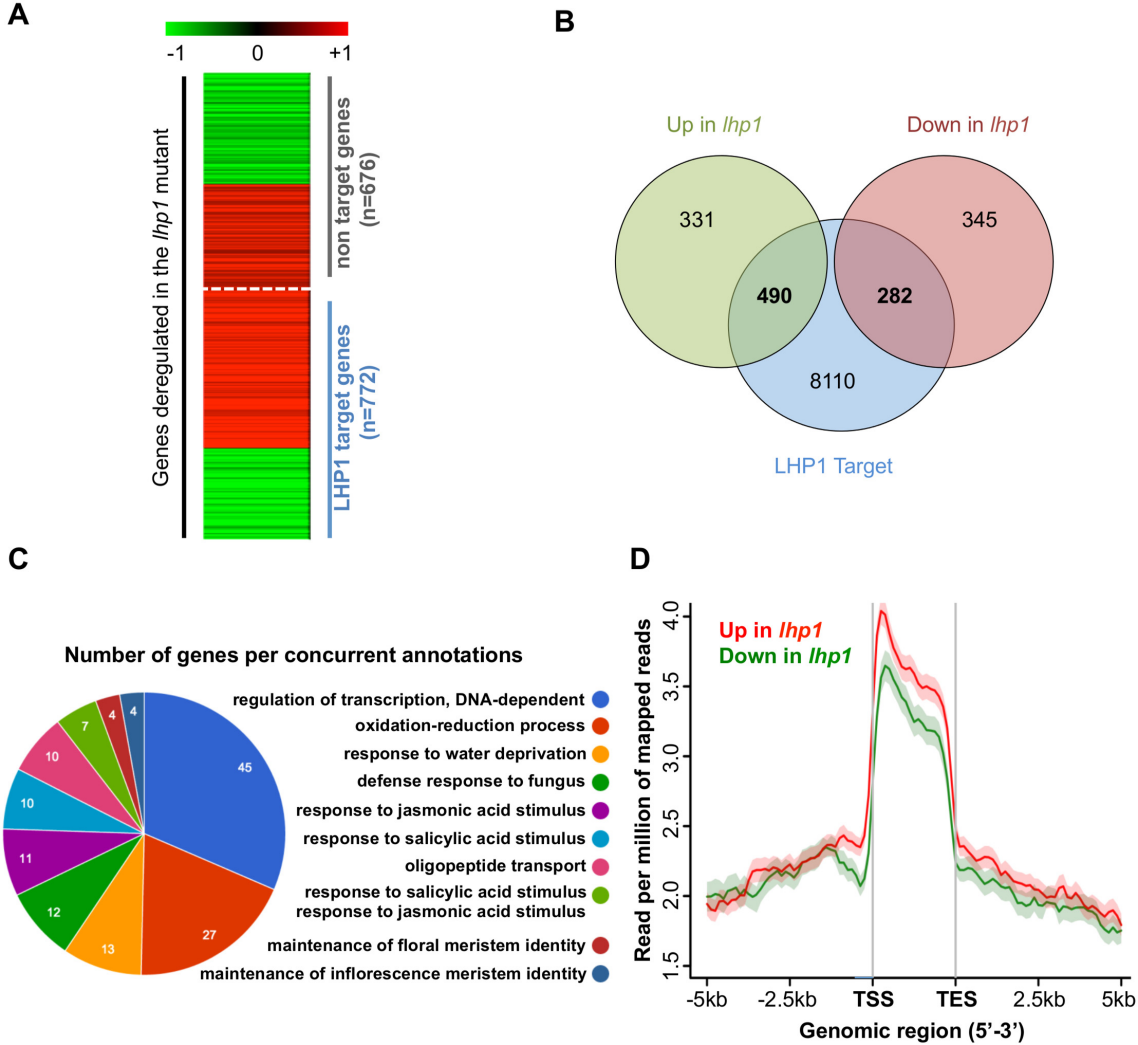
Relationship between the level of LHP1 binding and the magnitude of gene expression. **(A)** Heatmap showing fold change of expression level of LHP1-targeted and LHP1 unmarked genes in the *lhp1* mutant compared to WT (Genes with a log2 fold change of -1 or lower are coloured in green and genes with a log2 fold change of +1 or above are coloured in red). Only genes which are mis-regulated in *lhp1* are shown here. LHP1-targeted genes are predominantly up-regulated in the mutant *lhp1*. (**B)** Venn diagram showing the relationship of LHP1-targeted genes to gene expression. A higher number of LHP1-targeted genes are up-regulated in the mutant *lhp1*, compared to non-target genes. (**C)** Functional annotation of LHP1 targets which are up-regulated in the *lhp1*mutant (UT). (**D)** Average tag density profile of LHP1 on targeted and differentially regulated genes. Mean-normalized ChIP-Seq densities of equal bins along the gene and 2-kb region flanking the TSS or the TES were plotted. Up and down regulated genes are categorized with a p-value cutoff of 0.05 and fold change of one.

To better understand the role of LHP1 in gene expression regulation, the behavior of LHP1-target genes was assessed in *lhp1* mutant plants. Globally, those target genes exhibit a higher expression level in the *lhp1* mutant compared to WT (S1 Fig and S1 Appendix). However, the differential expression analysis of LHP1-target genes in *lhp1* versus WT (p-value <0.05) suggests that LHP1 could be both an activator and a repressor of transcription (Fig 2A). Indeed, more than one third of LHP1 target genes that were mis-regulated in the *lhp1* mutant were down-regulated (282 genes; Fig 2B; S2 and S3 Appendices). LHP1 Down-Regulated Targets (DT) in *lhp1* were preferentially involved in functions related to auxin response and environmental stimuli (S2 Fig). In contrast, Up-Regulated Targets (UT) are not particularly involved in any of these functions, but in the regulation of flower development, including several transcription factors, such as *CRABS CLAW* (*CRC*, AT1G69180), *FLORAL TRANSITION AT THE MERISTEM6* (*FTM6*, AT1G53160), *PETAL LOSS* (*PTL*, AT5G03680), *REPRODUCTIVE MERISTEM 35* (*ATREM35*, AT4G31615), *REPRODUCTIVE MERISTEM 1* (*ATREM1*, AT4G31610), *SEPALLATA3* (*SEP3*, AT1G24260), *SEPALLATA 4* (*SEP4*, AT2G03710), *AGAMOUS-LIKE 42* (AT5G62165), *PISTILLATA* (*PI*, AT5G20240), *MATERNAL EFFECT EMBRYO ARREST 24* (*MEE24*, AT2G34830) (Fig 2C and S3 Appendix). To determine how LHP1 could function both as a repressor and as an activator of gene expression, we asked whether its spatial distribution could differ between UT and DT genes. Interestingly, a comparison of the binding profiles of DT versus UT genes shows a lower global occupancy of LHP1 on DT with a more pronounced difference around the +1 nucleosomal position downstream of TSS (Fig 2D and S3 Fig), LHP1 being more present on this region of the UT genes. This differential and higher binding signal around the TSS of UT genes supports the idea that LHP1 recognizes its targets in a context-dependent manner. In addition, to that we also observed difference at the proximal promoter part suggesting a role of LHP1 in the chromatin accessibility of this specific region.

To better characterize the context-related binding of LHP1, we performed *de novo* motif analysis of DT and UT bound regions, in comparison to a prediction of all targets. The binding motif prediction of DT versus UT shows alternative consensus (S4 Fig). According to the JASPAR and AthaMap database of conserved motifs [44], DT genes exhibit a so-called core binding factor motif (CBF), which is part of the promoters of actively expressed genes. On the other hand, UT genes show an enrichment of the GATC tetranucleotide motif specific for HD2 binding (through HOX homeodomain). A second motif enriched in UT genes corresponded to the AC (Donor-acceptor splice site) in *Arabidopsis*. If we consider all LHP1 targets together for a prediction of consensus motifs, an alternative canonical motif (p value = 1e-35) can be found, although more degenerated (S4 Fig). According to JASPAR, this sequence corresponds to the element recognized by the ABI3 (ABSCISIC ACID-INSENSITIVE 3) transcription factor, involved in plant growth and development. It is worth noting however, that although the enrichment of these motives was highly significant, it was observed in only a relatively small subset of LHP1 target genes, indicating that additional factors play a prominent role in LHP1 recruitment. Altogether, our results suggest that LHP1 is recruited on these sequences but in a context-dependent manner to induce different outputs.

### Opposite profiles of H3K27me3 deposition in LHP1 targets can be identified in *lhp1* mutants

**Fig 3:**
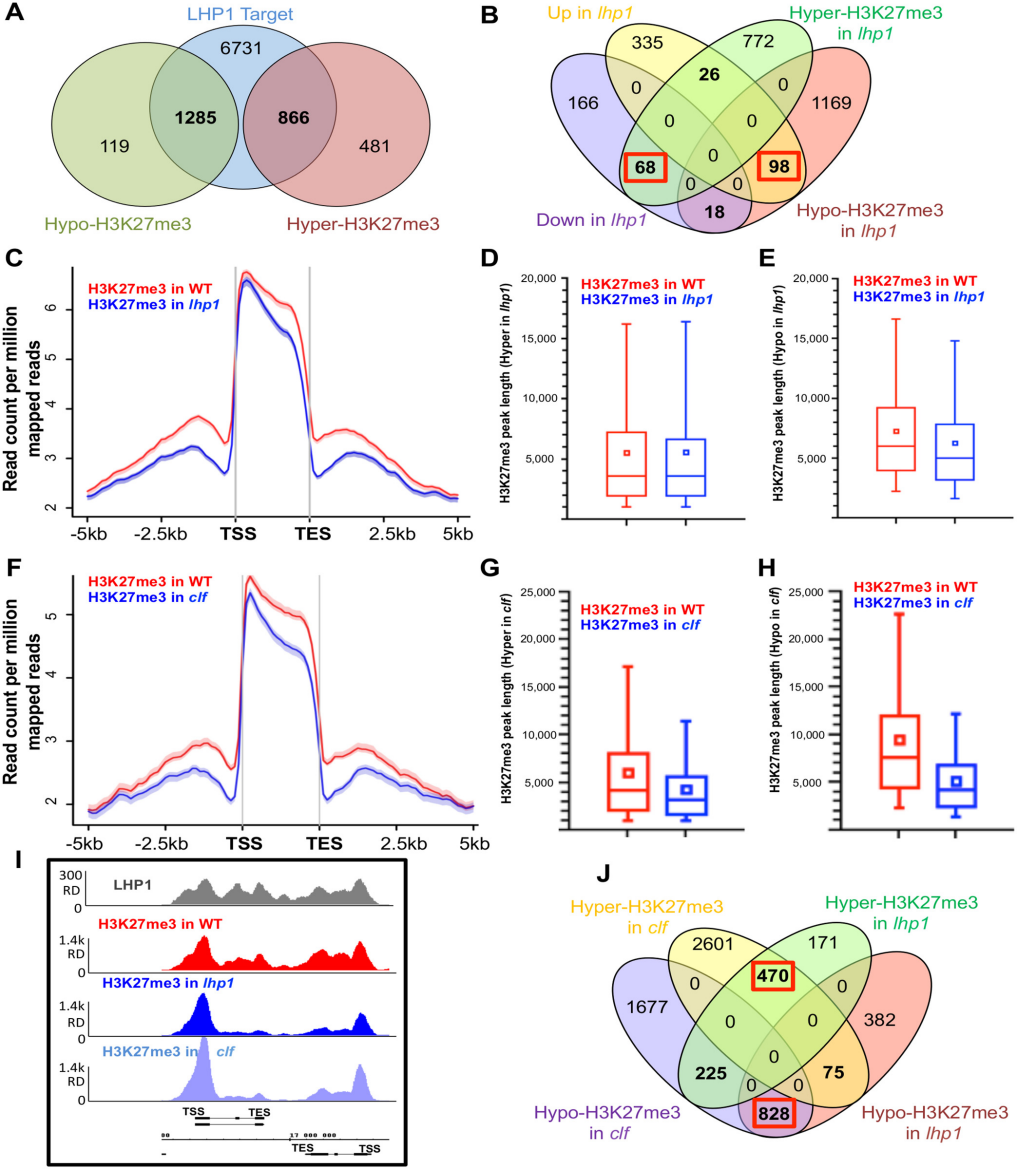
H3K27me3 spreading is affected in the *lhp1* and *clf* mutants. **(A)** Venn diagram showing differential marking of H3K27me3 deposition and LHP1 binding. Hyper (Higher enrichment) and Hypo-H3K27me3 (lower enrichment) refers to differential marking of H3K27me3 in the *lhp1* mutant compared to WT. (**B)** Venn diagram showing that Hyper-methylated (H3K27me3) genes are predominantly down-regulated in *lhp1* and Hypo-H3K27me3 genes are up-regulated in *lhp1*, as highlighted by the red boxes. (**C)** H3K27me3 distribution pattern (tag density) over the CDS and flanking regions for WT and *lhp1*. Mean-normalized ChIP-Seq densities of equal bins along the gene and 5 kb region flanking the TSS or the TES were plotted. (**D)** Boxplot showing differential peak lengths of H3K27me3 in WT and *lhp1* over Hyper-H3K27me3 region (Higher enrichment of H3K27me3 in *lhp1*, compared to WT). (**E)** Boxplot showing differential peak lengths of H3K27me3 in WT and *lhp1* over Hypo-H3K27me3 region (Lower enrichment of H3K27me3 in *lhp1*, compared to WT). (**F)** H3K27me3 distribution pattern (tag density) over the CDS and flanking regions for WT and *clf*. Normalizaton of coverage densities of equal bins using spline algorithm was performed over the genes and flanking 5 kb region. (**G)** Boxplot showing differential peak lengths of H3K27me3 in *clf* and WT over Hyper-H3K27me3 region (Higher enrichment of H3K27me3 in *clf*, compared to WT). (**H)** Boxplot showing differential peak lengths of H3K27me3 in *clf* and WT over Hypo-H3K27me3 region (Lower enrichment of H3K27me3 in *clf*, compared to WT). (**I)** LHP1 binding andH3K27me3 deposition in WT, *lhp1* and *clf* across two genes. Decreased level of H3K27me3 towards the 3’-end of both genes is observed in *lhp1* and *clf* compared to WT. (**J)** Venn diagram showing differential H3K27me3 deposition in *lhp1* and *clf*. A high overlapping of Hypo and Hyper-methylated genes can be observed between *lhp1* and *clf*, as indicated in the red boxes.

It is known that LHP1 binds H3K27me3 through its chromodomain [32–34], although the contribution of LHP1 to the genome-wide distribution of H3K27me3 remains unclear. Therefore, we performed two biological replicate of an H3K27me3 ChIP-Seq in both WT and *lhp1* mutant plants. Strikingly, our results show that the knock-out of LHP1 causes both a decrease and an increase of H3K27me3, among LHP1 targets. Considering a q-value cutoff of 0.05 and a fold change of 0.5 to identify significantly differentially marked genes in *lhp1* compared to WT, we determined that 64.29% (886/1347) of hyper-methylated genes (higher level of H3K27me3 in *lhp1* compared to WT) correspond to LHP1 targets (Fig 3A), whereas up to 91.52% (1285/1404) of hypo-methylated genes are targets of LHP1 (S4 and S5 Appendices). The number of TOM genes (TOM = LHP1 Targeted and H3K27me3 hypO-Methylated genes) are statistically significantly higher (Binomial test p-value< 2.2e-16) than TRM genes (TRM = LHP1 Targeted and H3K27me3 hypeR-Methylated genes). Our analyses indicate that the lack of LHP1 impacts H3K27me3 deposition on most loci harboring this histone mark, but unexpectedly leads to a dual effect on H3K27me3 deposition and/or maintenance. Two examples of crucial genes for flower development are given by *FLOWERING LOCUS C (FLC)* and *AGAMOUS* (*AG)*, exhibiting a decreased deposition of H3K27me3 across their gene bodies in the *lhp1* background (S5 and S6 Figs). Importantly, H3K27me3 deposition was restored at the *FLC* locus in complemented mutant lines (S5 Fig), confirming the direct role of LHP1 in this process. On the other hand, four genes from the YUCCA family (*YUC5*, *6*, *8* and *9*), involved in the biosynthesis of auxin, show an enhanced deposition of H3K27me3 in the *lhp1* mutants (S7 Fig). Remarkably, this effect on H3K27me3 correlates with their transcriptional behavior in the mutant background (S4 and S5 Appendices).

### LHP1 controls H3K27me3 spreading across the body of target genes

To determine the relationship between LHP1 binding, H3K27me3 levels and gene expression, we compared LHP1 targeted-H3K27me3 modified genes (TRM and TOM genes) with their expression levels in *lhp1* versus WT. We observed that hyper-methylated genes (TRM) are mainly down regulated (68 genes against 26; Exact Binomial test p-value = 8.658e-06), whereas hypo-methylated genes (TOM) are mostly up regulated (98 genes against 18; Exact Binomial test p-value = 8.246e-15) (Fig 3B). Altogether, LHP1 regulates H3K27me3 levels in opposite manners on different targets, impacting gene expression accordingly.

We then focused our analysis on TOM genes in *lhp1*, to elucidate the role of LHP1 in H3K27me3 deposition and/or maintenance. The comparison between H3K27me3 depositions in this sub-set of genes in *lhp1* versus WT revealed that H3K27me3 was not modified on the first nucleosomes after the TSS but was clear on the rest of the gene body (Fig 3C and S8 Fig). Furthermore, the differential deposition of H3K27me3 in *lhp1* turns more pronounced towards the 3’ end of the gene. This difference in H3K27me3 spreading was measured also by comparing the peak broadness in *lhp1* versus WT in TRM (Fig 3D) and in TOM (Fig 3E): the peak length did not differ on TRM between WT and *lhp1* (Two-sample t-test p-value = 0.81), whereas it was severely reduced on TOM genes in *lhp1* (Two-sample t-test p-value = 3.4e-12), suggesting that this effect is characteristic of TOM genes. Our results indicate that LHP1 is involved in spreading of H3K27me3 in plants, in a context-dependent manner.

We then asked which methyl-transferase could be responsible for the spreading of H3K27me3 on LHP1 target genes. The floral transition is induced by the PRC1 RING-finger protein AtRING1A, via its interaction with LHP1 and CLF [45] and AtRING1A interaction with LHP1 affects its binding to H3K27me3. In the same way, CLF-containing PRC2 complexes were shown to participate in the deposition of H3K27me3 [23]. Also, it was found that Multicopy Suppressor of IRA1 (MSI1), a PRC2 component necessary for H3K27me3 deposition, interacts with LHP1, triggering a positive feedback loop to recruit PRC2 to chromatin that carries H3K27me3 [39]. Hence, we performed two biological replicate of a ChIP-Seq of H3K27me3 in the PRC2-deficient *clf* mutant and compared the results with data described above. Profiling of H3K27me3 in *clf* revealed altered enrichment patterns across the gene body, similar to the ones observed in *lhp1* (Fig 3F), although the difference of H3K27me3 deposition in the TSS was more pronounced. Moreover, the effect on the peak broadness in *clf* in hyper-methylated genes and hypo-methylated genes (Student t-test p-value = 2.12e-229) is also comparable to *lhp1* (Fig 3E, 3G and 3H). The altered spreading in *lhp1* and *clf* is clearly illustrated in the example of Fig 3I. Furthermore, a high correlation could be established between hyper (Binomial test p-value < 2.2e-16) and hypo methylation (Binomial test p-value <2.2e-16) in the *lhp1* and *clf* mutants of LHP1 target genes (Fig 3J). Thus, our findings indicate the spreading of the mark towards the 3’ end of the gene is dependent on both CLF and LHP1.

### LHP1 shapes chromatin conformation in *Arabidopsis*

**Fig 4:**
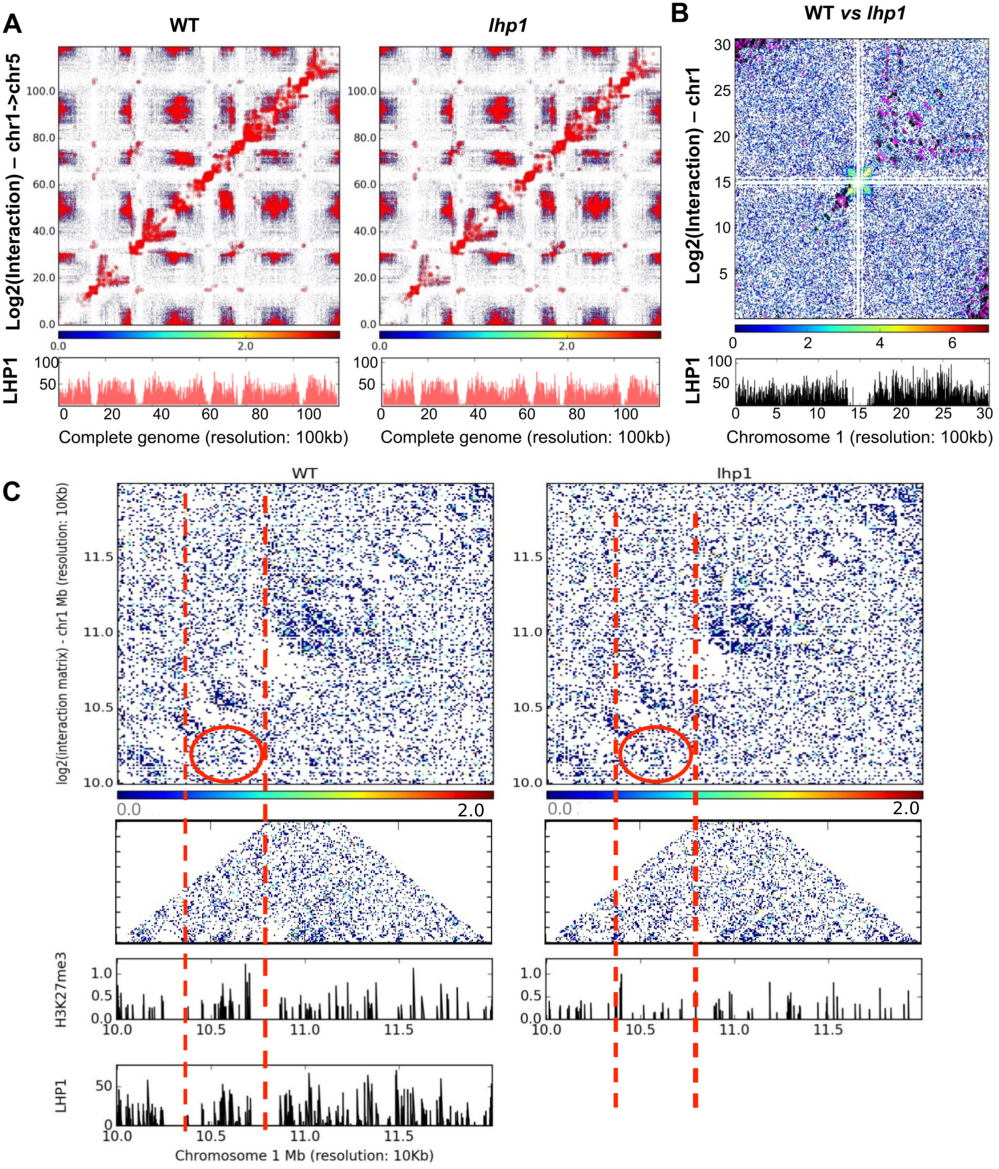
Genome topology is globally altered in the *lhp1* backgorund. **(A)** 2D interaction map showing significant interactions in WT and *lhp1*. Highly significant interactions are marked as red dots in their corresponding boxes. The color scale represents log2 (interaction) values. Lower panel in red (marked “LHP1”) are peaks from the LHP1 WT ChIP-seq. The two LHP1 panels are identical as they correspond to LHP1 binding in WT. The second LHP1 panel is shown here to correlate this dataset with the Hi-C in the mutant. (**B)** A screenshot of zoomed 2D interaction map showing intra-chromosomal interaction and LHP1 binding region for Chromosome 1. Centromeric interactions in those regions are masked. WT specific interactions are marked as black dots and *lhp1* specific interactions are marked as purple dots. The color scale represents the log2 (interaction), which is calculated against the background (taken as *lhp1* mutant). Lower panel in black (marked “LHP1”) shows the peaks of LHP1 deposition in WT ChIP-seq. (**C)** 2D interaction map showing the loss of interaction in *lhp1* when compared to the same region in WT (top panels). Lower panels show the loss of H3K27me3 in the same region. In red it is highlighted the region in WT where LHP1 and H3K27me3 co-occur, exhibiting interaction changes in *lhp1* mutants, along with the loss of H3K27me3.

LHP1 has been recently implicated in the dynamic conformation of a chromatin loop fine-tuning the promoter activity of a protein-coding gene, regulated by long noncoding transcription [14]. Using genome-wide chromatin conformation capture (Hi-C), we mapped the spatial contacts and distribution of genes in chromatin between different parts of the *Arabidopsis* genome for WT and *lhp1* plants. Digestion of the *Arabidopsis* genome by *Hind*III restriction enzyme produces fragments which range from small (< 1-kb) to large (> 10-kb) in size (S9 Fig). The differences of chromosome architecture were then correlated with LHP1 binding and gene expression. In general, we observed small local interactive domains across the genome, as described earlier [46]. We identified 50,230 interacting regions in the WT genome, based on 1-kb resolution binning and using binomial test with p-value cut-off of 0.05 (S7 Appendix). In *lhp1*, the total number of loci interactions with the same criteria was higher than in the WT, reaching a total of 70,830 (S7 Appendix). Our further analyses are based on 1-kb binning, although to depict as a 2D graph, we used a 100-kb resolution (Fig 4A). According to our Hi-C analysis, the chromosomal contact maps at 100-kb resolution show significant changes between WT and *lhp1* (Fig 4A and 4B). Also the changes in the interactions were seen in the pericentromeric regions distal to the centromere, as well as in telomeres (Fig 4B). Analysis of the intra chromosomal interactions reveal short distance (< 10kb) chromatin loops in both WT and *lhp1* plants, although the knockout of *LHP1* impacts the global interaction pattern (Fig 4C and S10 Fig). Interestingly, both gain and loss of LHP1-dependent intra- and inter-chromosomal interactions can be detected (S7 Appendix). We found that 17,006 interacting pairs are specific to WT.

**Fig 5:**
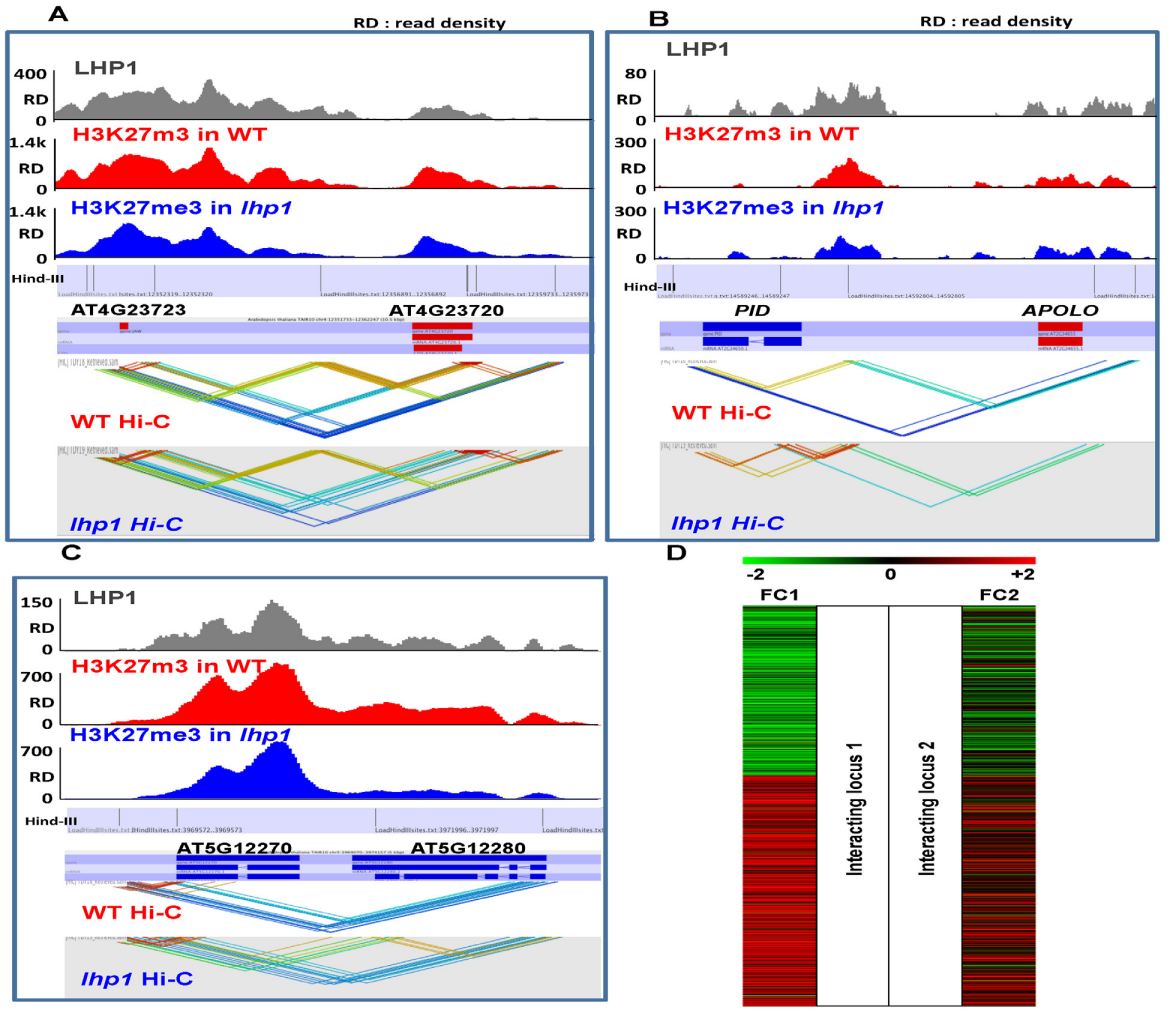
Global changes in chromatin interactions are observed in *lhp1*, impacting gene transcription. **(A)** Gene pairs showing altered chromatin interactions and reduced levels of H3K27me3 in *lhp1* compared to WT. **(B)** Hi-C interactions between the pair of gene loci *PID* and *APOLO* (chr2:14588900–14599067). Several interactions can be detected between these two loci in WT. These interactions are diminished and even lost (the one in dark blue) in the *lhp1* mutant. (**C**) Genome browser screenshot showing gene pairs revealing loss of chromatin interactions and reduced levels of H3K27me3 in *lhp1* compared to WT. For **A** to **C**, colors indicate different interactions (red to blue) in *cis* and in *trans* (not adjacent *Hind* III sites). (**D)** Expression level changes in*l hp1* compared to WT of significantly interacting pairs of genes which are LHP1-targeted. Colors were attributed according to the fold-change (FC) observed in the expression level: genes with a log 2 fold change of 2 or above are coloured in red while genes with a log 2 fold change of -2 or lower are coloured in green and genes with a log 2 fold change between -2 and 2 are in black. Interacting pairs show similar transcriptional behavior in the *lhp1* background.

Interestingly, 76.8% (13,069) of interacting regions that were significantly lost in the mutant background, are associated to LHP1 (in WT) and displayed a hypo-methylation of H3K27me3 in *lhp1* mutant (Fig 4C). Several gene pairs, including the known case of the *APOLO*-*PID* region [14], show an altered pattern of chromatin interactions (Fig 5A, 5B and 5C). The involvement of LHP1 in loop formation was further confirmed by demonstrating that it was restored in complemented mutant lines (S11 Fig). Altogether, these results suggest that LHP1 is a major determinant of chromatin architecture.

In order to evaluate the effect of LHP1-dependent chromatin interaction on gene transcriptional regulation, we profiled the interacting genes to their expression in WT versus *lhp1* lines. We considered all chromatin loops for which at least one of their ends is an LHP1 target, and we compared their transcriptional behavior in the *lhp1* mutant of the two genes, one at each end of the loop. Out of 887 loop-associated pairs in WT (LHP1 chip-marked and significantly expression-regulated), 667 pairs are affected in *lhp1*. Strikingly, the interacting pairs of genes exhibited a segregated co-expression pattern in the *lhp1* plants (Fig 5D and S12 Fig). Either the pairs are highly expressed or lowly expressed in the *lhp1* background compared to WT, suggesting a co-regulation of gene expression mediated by LHP1-dependent chromatin 3D conformation. Therefore, LHP1 acts at different levels on chromatin organization, thus shaping *Arabidopsis* genome topology and modulating gene expression.

## Discussion

Both in plants and animals, H3K27me3 constitutes a characteristic mark of developmental genes expressed in specific cell types [33, 34, 47]. In *Arabidopsis* seedlings, over 20% of protein-coding genes are marked by H3K27me3, including an important number of hormone and stress response genes [25, 34], suggesting a role of Polycomb complexes in plant developmental plasticity.

Genome-wide maps of H3K27me3 and/or the occupancy of PRC1 and 2 subunits are available in animals [48–51] as well as in plants [25, 33, 34, 52–58]. Two previous studies have addressed the characterization of LHP1 distribution across the *Arabidopsis* genome. First, Turck and coworkers (2007) performed a ChIP followed by the hybridization to an *Arabidopsis* Chromosome 4 tiling array (ChIP-chip), showing that LHP1 associates with hundreds of small domains, mostly corresponding to genes located within euchromatin. Furthermore, the authors showed that LHP1 association with chromatin highly correlates with domains marked by H3K27me3. A genome wide approach using a LHP1 tagged with a DNA methylation enzyme and tilling arrays was performed at the same time by Zhang and coworkers (2007), confirming that LHP1 co-localizes with H3K27me3 and that they interact *in vitro*. Based on this method, 2,354 regions bound by LHP1 genome-wide were identified. Since these findings, suggesting that LHP1 represents an equivalent of a component of PRC1 in plants, novel technologies have been developed. In our work, LHP1 ChIP followed by high throughput sequencing (ChIP-Seq) served to identify almost 14,000 peaks, corresponding to over 8,800 genes expanding previous datasets. Furthermore, by comparing these data to our H3K27me3 and RNA Pol II ChIP-Seq results, we could not only confirm the high correlation between LHP1 and H3K27me3 deposition, but also showed a clear anti-correlation between their presence and Pol II occupancy (Fig 1A–1C). Furthermore, we could establish the relationship between gene transcriptional activity and LHP1 binding (Fig 1E). Consistent with previously described function of LHP1 as a transcriptional repressor, we found that the majority of LHP1 targets were hypo-methylated in the *lhp1* mutant. This finding indicates that LHP1 not only recognizes H3K27me3 but can also influence the deposition of this mark. Previously, Turck and coworkers (2007) reported that H3K27me3 deposition was not modified in the *lhp1* background using a ChIP-chip analysis with the Chromosome 4 tiling microarray. This apparent discrepancy may be attributed to the approaches used: our results show a global reduction of H3K27me3 deposition, and fine mapping of H3K27me3 using ChIP-seq clearly reveals a specific reduction of H3K27me3 spreading. These differences would have been difficult to detect using ChIP-chip since the DNA fragments present on the micro-array are relatively large (between 0.3 and 1.2kb). Furthermore, our results are consistent with the work of Derkasheva and coworkers (2010) who observed a reduction of H3K27me3 at specific loci in roots of the *lhp1* mutant. It is known that the H3K27me3 repressive mark can often spread from the nucleation center into flanking regions [20, 23, 59]. In this work, we demonstrate that the spreading of this mark towards the 3’ end of the gene body is a common feature of LHP1 targets, depending on LHP1 and the PRC2 subunit CLF, whereas the deposition of H3K27me3 around the TSS is likely to recruit CLF (Fig 3C and 3F). Remarkably, an independent approach recently published by Wang and coworkers (2016), based on H3K27me3 ChIP-Seq and the previous reports of ChIP-chip [33], served to demonstrate that LHP1 and CLF participate in elongation of H3K27me3 mark. Furthermore, according to this work, the spreading of H3K27me3 is independent from the PRC1-catalytic core subunits BMI1 and RING1.

Crossing our LHP1 ChIP-Seq results with those of our RNA-Seq of *lhp1* seedlings vs WT, we determined that 772 genes out of the 8882 LHP1-targets are deregulated in the mutant (Fig 2B). This does not necessarily indicate that LHP1 binding exerts no function on gene expression regulation for the majority of its targets. It means that in control conditions, the transcriptional effect is detectable only for a subset of them, what would likely change in particular developmental contexts or in response to certain external stimuli, impacting the expression of different subsets of target genes, in particular cell types. Unexpectedly, transcriptional analyses of the *lhp1* mutants together with the H3K27me3 ChIP-Seq in the same background revealed that a subset of LHP1-targeted genes became hyper-methylated and down-regulated in *lhp1* mutants, suggesting that LHP1 can function as a positive regulator of a sub-set of genes. Considering that *lhp1* mutants have strong pleiotropic phenotypes, the downregulation of genes could be a consequence of secondary effects rather than a direct role of LHP1 as transcriptional activator. However, it has been shown by Rizzardi and coworkers (2011) that LHP1 exerts a positive role in transcriptional activation of the YUCCA genes, suggesting that LHP1 is globally a repressor of transcription but in some cases it can be an activator. Notably, the sub-set of down regulated genes in the *lhp1* mutant is enriched in genes involved in functions related to auxin response and environmental stimuli (Fig 2C). It was shown that an enrichment of H3K27me3 characterizes an important number of genes implicated in the biosynthesis, transport, perception, and signal transduction of auxin, suggesting a common control involving this mark of the entire signaling pathway [25]. Interestingly, *lhp1* plants exhibit a reduced rate of auxin biosynthesis, which correlates with the down-regulation of specific genes involved in this pathway, a sub-set of the YUCCA genes [60]. It was shown that LHP1 can dynamically target a number of the YUCCA genes in an auxin-dependent fashion affecting their transcriptional activity, demonstrating the role of LHP1 in auxin signaling. Here, we show that this deregulation is correlated with enhanced deposition of H3K27me3 across their gene bodies (S7 Fig). In addition, LHP1 binding to the *APOLO* noncoding RNA locus is modulated by exogenous auxin, impacting the transcription of its neighboring gene [14]. However, in the latter case, LHP1 functions as a transcriptional repressor, as expected. The molecular mechanisms underlying its dual role on gene expression remain to be elucidated.

A classification of up and down-regulated targets in the *lhp1* mutants allowed us to identify a differential LHP1-binding pattern for each of them, being more abundant on the first nucleosomes on genes that it negatively regulates (Fig 2D). Moreover, alternative consensus motifs were found for each subset of target genes, suggesting that a context-dependent mechanism involves LHP1 and can lead to regulate gene expression in different ways (S4 Fig). So far, the identification of one single consensus sequence associated to the general recruitment of PRC has not been achieved. However, it was recently shown that PRC2 binding sites in *Arabidopsis* could contain putative GAGA factor binding motifs within the gene body, which may serve as recruiting elements or to strengthen the interaction of PRC2 at the target [53]. For some genes in particular, it was shown that the initiation of H3K27me3 deposition is regulated by *cis*-elements required and sufficient to recruit PRC2. This is the case of *FLC* [53, 59, 61, 62], *WUSCHEL (WUS)* [50] and *AGAMOUS* (*AG*) [23, 63], among others [21, 64–66]. Very recently, Wang and coworkers searched for consensus of H3K27me3 marked genes, whose expression is affected in the *lhp1* and *clf* mutants. Coincident to our results, a conserved motif, also enriched among all H3K27me3 marked genes, is the ABI3 element (S8 Appendix). Here, we identified alternative consensus motifs based on a more comprehensive list of LHP1 targets and depending on the sub-set of target genes considered (S4 Fig), i.e. transcriptionally up and downregulated in the *lhp1* mutant. Although not extensively present in each sub-set of genes, some of these motifs are found in up to 4.51% of DTs (the CBF motif) and 11.65% of UTs (the AC motif). Even though little is known about these motifs, which have been linked to various molecular mechanisms not necessarily related to LHP1 [44], the observation that alternative motifs are enriched depending on the sub-set of genes differentially regulated by LHP1, strongly suggests that the positive or negative regulation of gene expression by LHP1 depends on specific protein partners. Several LHP1 interactors have already been identified. For instance, we can mention the ribonucleoprotein LHP1-INTERACTINGFACTOR2 (LIF2) [67] and the cyclophilin protein AtCYP71 [68], although it remains unclear how these proteins affect LHP1 function and activity, and whether the interaction with these factors could modulate the outcome of LHP1 binding and its effect on gene expression. Also the plant-specific protein EMF1 interacts with LHP1 and a group of H3K4me3 demethylases [69]. Genome-wide localization of EMF1 coincides with H3K27me3, which is strongly affected in the *emf1* mutants. Furthermore, EMF1 is required for the repression of many PRC2 targets but also of other genes [70–72]. It has been shown that EMF1 and LHP1 interact with both PRC1 (AtRING1/AtBMI1) and PRC2 subunits [39, 69], suggesting the close cooperation of plant PRCs.

In addition to unraveling a role of LHP1 as a potential activator of gene expression, our work reveals a role in the modulation of global chromatin architecture. A number of previous studies provide evidence for a role of interactions between PRC components and long noncoding RNAs in the modulation of epigenetic regulation of gene expression [73]. For example, the *Arabidopsis* PRC2 subunit CLF binds the lncRNA *COLDAIR* in the repression of *FLC* after vernalization [74]. Likewise, it has recently been shown that LHP1 recognizes *in vivo* the long intergenic noncoding RNA (lincRNA) *APOLO* in the modulation of a dynamic chromatin loop encompassing the promoter of the *APOLO* neighboring gene, *PINOID (PID)* [14]. In *lhp1* mutants, the chromatin loop suffers a delayed formation after auxin-triggered opening, affecting *PID* transcription. Other animal chromodomain-containing proteins have been shown to bind ncRNAs, including the LHP1 closest homolog, HP1 [75, 76]. However, the global impact of LHP1 on the determination of chromatin topology was not yet elucidated. Here we made use of a Hi-C approach to show at genome-wide scale that the conformation of short-range loops is affected in *lhp1* mutants. Consistent with our previous work, the chromatin loop between *APOLO* and *PID* was also detectable in our Hi-C approach (Fig 5B), and we detected a significant alteration of the interacting chromatin profile across the whole locus in *lhp1*, thereby validating our previous gene-specific results. Both gain and loss of LHP1-dependent intra- and inter-chromosomal interactions have been detected. Several regions where significant loss of interactions was found in *lhp1* plants are associated with LHP1 targets and a decrease of H3K27me3. Strikingly, the correlation of the regulation of genes located in each end of the loop indicates that LHP1 plays a major role in all levels of genome organization to coordinate gene expression. In animals, growing evidence suggests that Polycomb complexes control spatial genome organization establishing regulatory contacts between distant loci, such as promoters and enhancers or functionally related genes [77–81]. It has been proposed that higher-order genome organization mediated by Polycomb complexes, notably PRC1, can maintain genes in three-dimensional interaction networks in a silent state, and the selective release of sub-sets of genes may determine cell fate decisions leading to organogenesis and development [80]. Genome-wide analyses of chromatin conformation in *Arabidopsis* WT and mutant lines have recently begun to shed light on plant genome topology features [46, 69, 82, 83]. Remarkably, Feng and coworkers (2014) showed by a Hi-C approach on the *clf*-*swn* double mutant that the interaction of domains consisting of clustered H3K27me3 genes was dramatically reduced or eliminated in the double mutant background, suggesting that H3K27me3 may act directly or indirectly to regulate the interactivity of these regions. In our work, we show not only that the LHP1-dependent interactions correlate with a decrease of H3K27me3, but remarkably that there exists a co-regulation of transcription of distant genes brought together by LHP1-dependent chromatin interactions. Hence, LHP1 determines proper expression patterns in plant development through the control of genome topology. Future work on the interactions between LHP1, CLF and other PRC subunits and long noncoding RNAs will allow gaining a comprehensive understanding of the determination of dynamic chromatin compaction and genome topology.

## Materials and Methods

### Plant material and growth conditions

*Arabidopsis thaliana* Wild Type (WT) were Columbia-0 (Col0), and seeds of *lhp1* (SALK_011762 line) and *clf* (SALK_N521003) mutants were in the same background. Arabidopsis *lhp1* mutant plants complemented with the *ProLHP1*:*LHP1*:*GFP* have been previously described [31]. Plants were grown in chambers at 20°C on sterile half-strength MS medium and 0.8% agar under long-days (16h of light at 20°C, 8h of darkness at 18°C). Seeds were surface-sterilized by treatment with bayrochlore for 20 min, washed, and imbibed in sterile-water for 2–4 days at 4°C to obtain homogeneous germination.

### RNA-seq assay

Total RNAs were extracted from 180 mg of shoots of 12-day-old seedlings with the ZR Plant RNA MiniPrep kit (Zymo Research), according to the manufacturer's instructions. HiSeq 50bp singleton reads from RNA-Seq were first adaptor trimmed and then analyzed using the TopHat and Cufflinks software. TopHat v2.0.9 [84] was utilized for alignment of short reads to the *Arabidopsis thaliana* genome TAIR10, Cufflinks v2.2.0 [85] for transcript assembly and differential expression, and cummeRbund v2.0.0 for visualization of differential analysis. Default parameters (p-value: 0.05; statistical correction: Benjamini Hochberg; FDR: 0.05) were used. A cutoff of 0.5 fold up- or down-regulation has been chosen to define differential expression.

### ChIP-seq assay

ChIP-seq assays were performed on 14-day-old shoot seedlings grown in plates using anti-GFP (Clontech 632592), anti-H3K27me3 (Millipore 07–449) or anti-RNA Pol II (Abcam ab817), modified from [86]. Five grams of shoot plantlets were cross-linked in 1% (v/v) formaldehyde at room temperature for 15mn. Crosslinking was then quenched with 0.125 M glycine for 5 min. The crosslinked plantlets were ground and nuclei were isolated and lysed in Nuclei Lysis Buffer (1% SDS, 50mM Tris-HCl pH 8, 10mM EDTA pH 8). Cross-linked chromatin was sonicated using a water bath Bioruptor UCD-200 (Diagenode, Liège, Belgium) (15s on/15s off pulses; 15 times). The complexes were immunoprecipitated with antibodies, overnight at 4°C with gentle shaking, and incubated for 1 h at 4°C with 40 μL of Protein AG UltraLink Resin (Thermo Scientific). For anti-GFP and anti-RNA Pol II immunoprecipitations, the beads were washed for 6 × 5 min in ChIP Dilution Buffer (1,1% Triton X-100, 1.2 mM EDTA pH 8, 16.7 mMTris-HCl pH 8 and 167 mMNaCl) and twice in TE. For anti-H3K27me3 immunoprecipitation, the beads were wash 2 × 5 min in ChIP Wash Buffer 1 (0.1% SDS, 1% Triton X-100, 20 mMTris-HCl pH 8, 2 mM EDTA pH 8, 150 mMNaCl),2 × 5 min in ChIP Wash Buffer 2 (0.1% SDS, 1% Triton X-100, 20 mMTris-HCl pH 8, 2 mM EDTA pH 8, 500 mMNaCl), 2 × 5 min in ChIP Wash Buffer 3 (0.25 M LiCl, 1% NP-40, 1% sodium deoxycholate,10 mMTris-HCl pH 8, 1 mM EDTA pH 8) and twice in TE (10 mMTris-HCl pH 8, 1 mM EDTA pH 8). ChIPed DNA was eluted by two 15-min incubations at 65°C with 250 μL Elution Buffer (1% SDS, 0.1 M NaHCO_3_). Chromatin was reverse-crosslinked by adding 20 μL of NaCl 5M and incubated over-night at 65°C. Reverse-cross-linked DNA was submitted to RNase and proteinase K digestion, and extracted with phenol-chloroform. DNA was ethanol precipitated in the presence of 20 μg of glycogen and resuspended in 50 μL of nuclease-free water (Ambion) in a DNA low-bind tube. 10 ng of IP or input DNA was used for ChIP-Seq library construction using NEBNext^®^ Ultra DNA Library Prep Kit for Illumina^®^ (New England Biolabs) according to manufacturer’s recommendations. For all libraries, twelve cycles of PCR were used. The quality of the libraries was assessed with Agilent 2100 Bioanalyzer (Agilent), and the libraries were subjected to 1 x 50 bp high-throughput sequencing by HiSeq2500 (Illumina) at IGBMC Microarray and Sequencing Platform (Illkirch).

### Hi-C assay

Five grams of 14-day-old shoot seedlings were cross-linked in 1% formaldehyde at room temperature for 15 min. Crosslinking was then quenched with 0.125 M glycine for 5 min. The cross-linked plantlets were ground and nuclei were isolated, washed with 1.2 x CutSmart^®^ Buffer (New England Biolabs) and resuspended in 500 μL of 1.2 x CutSmart^®^ Buffer. Then, 7.5 μL of 20% SDS was added and incubated at 65°C for 30 min followed by 30 min at 37°C with occasional mixing. SDS was sequestered by adding 50 μL of 20% Triton X-100 and incubating at 37°C for 60 min with occasional mixing. Next, 400U of HindIII-HF (New England Biolabs) was added and incubated at 37°C for overnight. To label the digested DNA ends, 1.5 μL of 10 mMdCTP, 1.5 μL of 10 mMdGTP, 1.5 μL of 10 mMdTTP, 37.5 μL of 0.4 mM biotin-14-dATP (Invitrogen) and 50 U of Klenow (New England Biolabs) were added and incubated at 37°C for 45 min with occasional mixing. To inactivate enzymes, 105 μL of 10% SDS were added and incubated at 65°C for 30 min with occasional mixing. Digesting chromatin mixture was transferred in ligation mix (750 μL of 10x T4 DNA ligase reaction buffer (New England Biolabs), 75 μL of 100 x BSA (New England Biolabs), 5.2 mL of nuclease-free water) and SDS was sequestered by adding 750 μL of Triton X-100 and incubating at 37°C for 60 min with occasional mixing. To ligate the biotin-labelled DNA ends, 50 U of T4 DNA ligase (Thermo Scientific) were added and incubated at 16°C for 4 hours. Crosslinks were reversed and proteins were degraded by adding 500 μg of proteinase K (Invitrogen) and incubating overnight at 65°C. Then, DNA was purified by performing twice phenol/chloroform extractions and precipitated using ethanol. Ethanol precipitated DNA samples were resuspended in nuclease-free water and RNAs were digested by adding 100 μg RNAse A (Qiagen) and incubating at 37°C for 30 min. To remove biotin from the unligated ends, 5 μg of DNA was mixed with 10 μg of BSA, 10 μL of 10x NEBuffer 2 (New England Biolabs), 1 μL of 10 mM dGTP and 5 U of T4 DNA polymerase (New England Biolabs) and incubated at 12°C for 2 hours. The reaction was stopped by adding 2 μL of 0.5 M EDTA pH 8. DNA was purified by performing phenol/chloroform extraction and ethanol precipitation. Then, DNA was resuspended in 100 μL of nuclease-free water and was sheared to a size of 300–500 basepairs using Bioruptor Plus (Diagenode). Sheared DNA ends was repaired by adding 14 μL of 10x ligation buffer (New England Biolabs), 14 μL of 2.5 mM dNTP mix (Invitrogen), 5 μL of T4 DNA polymerase (New England Biolabs), 5 μL of T4 polynucleotide kinase (New England Biolabs) and 1 μL of T4 Klenow (New England Biolabs) and incubating at room temperature for 30 min. DNA was purified with QiagenMinElute column (Qiagen) according to manufacturer’s recommendations and was eluted twice with 15 μL of TLE (10 mMTris pH 8, 0.1 mM EDTA). Then, a dATP was attached to the 3’ ends of the end-repaired DNA by adding 5 μL of NEBuffer 2, 10 μL of 10 mMdATP and 3 μL of Klenow (exo-) (New England Biolabs) by incubating at 37°C for 30 min. Then DNA was loaded in a 1% agarose gel and DNA fragments between 300 and 500 base pairs were purified with a Qiagen gel extraction kit (Qiagen). Biotin-labeled Hi-C DNA was bound to Dynabeads M-280 Streptavidin (Lifes Technologies) magnetic beads by incubating in 1 x No Tween Buffer (NTB: 5 mMTris H-Cl pH 8, 0.5 mM EDTA pH 8, 1 M NaCl) at room temperature for 45 min. Beads were washed twice in 1 x NTB and resuspended in 50 μL 1 x ligation buffer with PEG (Invitrogen). To ligate Illumina Paired End adapters to biotin-labeled Hi-C DNA bound beads, 6 picomoles of Paired End adapters (Illumina) and 1200 U T4 DNA Ligase (New England Biolabs) were added to a tube and incubated at room temperature for 2 hours. Hi-C DNA bound beads were washed three times with NTB and 0.1% Tween and resuspended in 23 μL of nuclease-free water. Then, Hi-C DNA libraries were amplified by adding 1 μL of PCR Primer PE 1.0 (Illumina), 1 μL of PCR Primer PE 2.0 (Illumina) and 25 μL of Phusion^®^ High-Fidelity DNA Polymerase (New England Biolabs) with the following program (98°C for 30 sec, 18 cycles of [98°C for 10 sec, 65°C for 30 sec, 72°C for 30 sec], 72°C for 5 min). After amplification, streptavidin-coated beads were removed and Hi-C libraries were purified with AgencourtAMPure XP magnetic beads (Beckman Coulter) and eluted in 21 μL of nuclease-free water. The quality of the libraries was assessed with Agilent 2100 Bioanalyzer (Agilent), and the libraries were subjected to 2 x 50 bp paired-end high-throughput sequencing by HiSeq 2500 (Illumina) at IGBMC Microarray and Sequencing Platform (Illkirch).

### Computational analysis of ChIP-seq

Single-end sequencing of ChIP samples was performed using Illumina GAIIx with a read length of 50 bp. Reads were quality controlled using FASTQC (http://www.bioinformatics.babraham.ac.uk/projects/fastqc/). Trimmomatic was used for quality trimming. Parameters for read quality filtering were set as follows: Minimum length of 36 bp; Mean Phred quality score greater than 30;Leading and trailing bases removal with base quality below 3; Sliding window of 4:15. The reads were mapped onto the TAIR10 assembly using Bowtie [87] with mismatch permission of 1 bp. Unique mapping of reads was adopted. To identify significantly enriched regions, we used MACS2 [88]. Parameters for peaks detection were set as follows: Number of duplicate reads at a location:1; Bandwidth:300; mfold of 5:30; q-value cutoff:0.05. Visualization and analysis of genome-wide enrichment profiles were done with IGB. Peak annotations such as proximity to genes and overlap on genomic features such as transposons and genes were performed using HOMER. SeqMINER was used for quantitative clustering based on tag density using a Density Array method with a wiggle window of 50 bp. NGSplot was used to profile the enrichment of this mark at transcriptional start sites (TSSs) and along the gene [89]. To identify regions that were significantly enriched in the histone modification H3K27me3, we used SICER [90] with parameters of W:200, G:600 for H3K27me3. RNA Pol II occupancy and shift between the WT and the *lhp1* mutant were deduced from the sequencing data using MACS2. DiffReps (settings: Window size 1kb; step size:100bp; P-value: 0.0001; Statistical testing method: Chi-square method) was used to find the differential marking between two histone modifications [91]. Spatial binding of the two peaks of LHP1 and H3K27me3 were done by Position-wise comparison using binning approach and plotted in hexplot. *De novo* motif analysis of LHP1 binding regions were screened for specific DNA motifs using HOMER [92]. The binding regions of the significantly up-regulated genes and down-regulated genes were analyzed. These significantly enriched motifs were compared with motif databases JASPAR [44].

### Computational analysis of Hi-C data

The Illumina paired end raw data was quality checked using FASTQC. The raw reads with adapter sequences and low quality bases were removed. Genome was split into compartments of Hind III fragments and further reads are mapped around the fragment end-points (S9 Fig). The processed high quality reads with more than 70% bases having Phred score greater than 30 were considered significant for further downstream analysis. The processed reads were then aligned against the reference database using HICUP [93]. HICUP receives processed FASTQ data which is then mapped against a reference genome and filtered to remove frequently encountered experimental artifacts. It produced paired read files in SAM/BAM format, each read pair corresponding to a putative Hi-C di-tag. The sam file generated was further used for analysis using HOMER [92]. HOMER searched for pairs of loci that have a greater number of Hi-C reads that is expected by chance, referred to as a significant interaction with p-value less than 0.2 and z-score greater than >0. The reads statistics related to truncation, filtration, mapping, deduplication are in S6 Appendix. These interacting loci are annotated with gene if it lies within 2kb region. Intra-chromosomal and inter-chromosomal interactions were analyzed and circos plots were drawn using CIRCOS tool.

## Supporting Information

S1 Appendix List of LHP1 target genes. TAIR10 Gene accession list marked by LHP1.
(XLSX)

S2 Appendix List of up- and down- regulated genes in *lhp1*. Up and down regulated TAIR10 Gene accession list.
(XLSX)

S3 Appendix List of LHP1 target genes deregulated in *lhp1*. TAIR10 Gene accession list marked by LHP1 and de-regulated by expression.
(XLSX)

S4 Appendix List of genes that are H3K27me3 hypo- and hyper- methylated in *lhp1*. TAIR10 Gene accession list which are methylated (hypo and hyper-methylated).
(XLSX)

S5 Appendix List of LHP1 target genes that are H3K27me3 hypo- and hyper-methylated in *lhp1*. TAIR10 Gene accession list marked by LHP1 and methylated (hypo and hyper-methylated).
(XLSX)

S6 Appendix The reads statistics, truncation, filtration, mapping, deduplication results from Hi-C experiments. Sequencing information of Hi-C data.
(XLSX)

S7 Appendix A. WT and B. *lhp1* interacting loci. Genome co-ordinate pairs representing the interacting loci derived from Hi-C analysis.
(XLSX)

S8 Appendix Full list of motifs found based on all LHP1 target genes. Sequence motifs detected from HOMER analysis of LHP1 target genes.
(PDF)

S1 Fig LHP1 targets behavior in *lhp1* versus WT. Box plot representing the expression level of LHP1 targeted genes in WT and *lhp1* mutant. Expression levels are depicted in RPKM (Read Per Kilobase per Million reads) that was derived using RNA-Seq.
(EPS)

S2 Fig Functional annotation of genes which are LHP1-targeted and down-regulated in the *lhp1* mutant (DT). Scatterplot (generated from Revigo) showing the functional categories of Down-regulated LHP1-targeted genes (DT). Biological process terms from GO are positioned in the semantic space. Semantic space refers to the closeness of the function (cluster of GO terms). This two dimensional space derived by multidimensional scaling to a matrix of the GO terms' semantic similarities. Highly enriched terms include Auxin biosynthesis, chromatin assembly and defense response to environmental stimuli. Enrichment of particular terms is given as color within the bubble. Size of bubble indicates the frequency of the GO term in the underlying TAIR10 gene ontology.
(EPS)

S3 Fig Average tag density profile of LHP1 on targeted and differentially regulated genes. The blue line represents the position of the first nucleosome after the TSS.
(EPS)

S4 Fig Enriched nucleotide motifs in LHP1-binding regions across the *Arabidopsis* genome. (**A**) HOMER nucleotide motif enrichment in the peaks overlapping the down-regulated LHP1-targeted genes (DT), using a significant p-value of 0.05. These motifs are later annotated against a known motif database (JASPAR). Motifs derived (JASPAR:POL008.1) from DT peaks are annotated DCE (Downstream core element) elements which are transcribed by RNAp PolII. (**B**) Nucleotide pattern in LHP1 peaks associated with the up-regulated LHP1-targeted genes (UT). UT peaks shows DCE motif; AC motif (JASPAR:SD0002) which are specific to genomic splice sites; TRP(MYB) motif: transcription factor binding site. (**C**) *De novo* motif discovery identifies CGTTCATG in genome-wide LHP1 binding site. This pattern is found exactly in the midpoint of the LHP1 ChIP-Seq peaks (LHP1 ChIPseq peaks are broad and this candidate sequence pattern is at their center).
(EPS)

S5 Fig Co-marking of LHP1 and H3K27me3 across the *FLC* locus; Reduced levels of H3K27me3 in *lhp1* and *clf*. **(A)** Genome browser screenshot showing tag density of LHP1, as well as H3K27me3 over the *FLC* gene in WT, *lhp1* and *clf*. (**B)** Schematic representation of the regions of the *FLC* locus analysed in **C**. Black boxes correspond to exons, the arrow indicates the site of translation initiation, numbers indicate the position of primer pairs used (S13 Fig) [94] (**C)** Quantification data of the chromatin immunoprecipitation results. Nuclei were extracted from 10-day-old seedlings grown under LD and immunoprecipitation was performed with antibodies specific for H3K27me3. Average relative quantities ± sd are shown for each sample.
(EPS)

S6 Fig Co-marking of LHP1 and H3K27me3 across the *AG* locus; Reduced levels of H3K27me3 in lhp1 and clf. Genome browser screenshot showing tag density of LHP1, as well as H3K27me3 over the *AG* gene in WT, *lhp1* and *clf*.
(EPS)

S7 Fig Profiles of LHP1 binding and H3K27me3 deposition across YUCCA genes. Genome browser screenshot showing profile patterns of LHP1, H3K27me3 over the YUCCA genes *YUC5*, *6*, *8* and *9* in WT and *lhp1*.
(EPS)

S8 Fig H3K27me3 distribution pattern (tag density) over the TSS for WT and *lhp1*. The green line represents the position of the first nucleosome after the TSS.
(EPS)

S9 Fig Fragment length histogram generated from the *Arabidopsis*genome, by *Hind* III restriction enzyme digestion. Histogram showing that 1-kb fragments are large in number, validating that 1-kb resolution based binning is possible using *Hind*III as a restriction enzyme for Hi-C.
(EPS)

S10 Fig Hi-C interactions are diminished in *lhp1* compared to WT. HiC interaction differences in a region (chr1:16mb– 17.5mb). Several interactions can be detected along the loci in WT. These interactions (highlighted between red dotted lines) are reduced and even lost (the one in blue) in the *lhp1* mutant. The color code in the interactome (blue to red) represents the number of significant interactions.
(EPS)

S11 Fig LHP1 regulates gene loop formation. Relative loop conformation measured by BglII-3C-qPCR, considering the WT level as 100% [14]. Error bars represent the standard deviation of three biological replicates.
(EPS)

S12 Fig Positive correlation of gene expression between two LHP1-targeted genes from the same interacting pair in WT. Scatterplot showing positive correlation of gene expression between two LHP1-targeted interacting pairs of genes in WT. These are significantly interacting pairs derived from the Hi-C experiment in WT.
(EPS)

S13 Fig List of oligonucleotides used for qPCR. Sequences used for qPCR analysis.
(EPS)
