# Supplementary material for: LHP1 Regulates H3K27me3 Spreading and Shapes the Three-Dimensional Conformation of the Arabidopsis Genome
Source: PLoS One. 2016 Jul 13;11(7):e0158936. doi: 10.1371/journal.pone.0158936 (PMC4943711; doi:10.1371/journal.pone.0158936)
Supplement: S8 Appendix — Sequence motifs detected from HOMER analysis of LHP1 target genes. (PDF) [file pone.0158936.s008.pdf]

Homer *de novo* Motif Results (TDJG22TvsTDJG21C_MotifResults/)

Known Motif Enrichment Results
Gene Ontology Enrichment Results
If Homer is having trouble matching a motif to a known motif, try copy/pasting the matrix file into STAMP
More information on motif finding results: HOMER | Description of Results | Tips
Total target sequences = 13892
Total background sequences = 35830
* - possible false positive

Rank	Motif	P-value	log P-pvalue	% of Targets	% of Background	STD(Bg STD)	Best Match/Details	Motif File
1	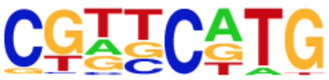	1e-35	-8.108e+01	16.43%	12.77%	55.9bp (59.9bp)	MA0564.1_ABI3/Jaspar(0.650) More Information | Similar Motifs Found	motif file (matri
2	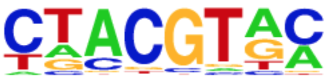	1e-28	-6.604e+01	18.02%	14.56%	57.2bp (62.6bp)	O2(bZIP)/Zea mays/AthaMap(0.700) More Information | Similar Motifs Found	motif file (matri
3	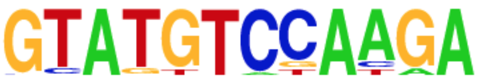	1e-22	-5.147e+01	0.15%	0.01%	51.6bp (0.0bp)	AtSPL3(SBP)/Arabidopsis thaliana/AthaMap(0.505) More Information | Similar Motifs Found	motif file (matri
4	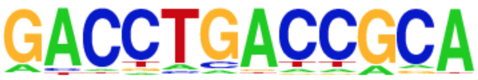	1e-22	-5.136e+01	0.19%	0.01%	55.2bp (41.1bp)	HVH21(HD-KNOTTED)/Hordeum vulgare/AthaMap(0.588) More Information | Similar Motifs Found	motif file (matri
5	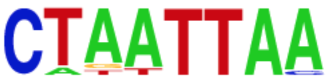	1e-21	-5.008e+01	10.70%	8.34%	56.9bp (59.5bp)	MF0010.1_Homeobox_class/Jaspar(0.678) More Information | Similar Motifs Found	motif file (matri
6	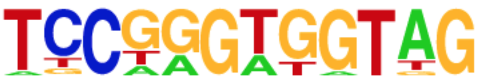	1e-21	-4.998e+01	0.12%	0.00%	47.7bp (0.5bp)	AtMYB84(MYB)/Arabidopsis thaliana/AthaMap(0.612) More Information | Similar Motifs Found	motif file (matri
7	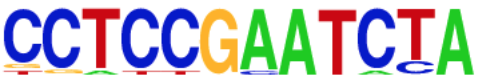	1e-20	-4.620e+01	0.12%	0.00%	60.1bp (36.7bp)	MA0121.1_ARR10/Jaspar(0.647) More Information | Similar Motifs Found	motif file (matri
8	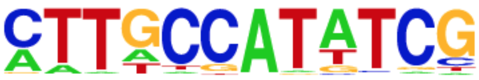	1e-20	-4.620e+01	0.12%	0.01%	50.2bp (0.0bp)	MA0082.1_squamosa/Jaspar(0.603) More Information | Similar Motifs Found	motif file (matri
9	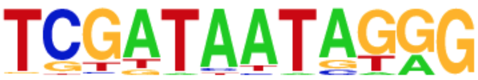	1e-18	-4.159e+01	0.37%	0.08%	59.8bp (52.1bp)	ATHB5(HD-ZIP)/Arabidopsis thaliana/AthaMap(0.572) More Information | Similar Motifs Found	motif file (matri
10	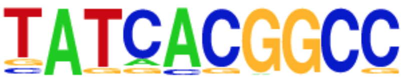	1e-17	-4.144e+01	0.21%	0.02%	50.0bp (41.6bp)	ABI4(1)(AP2/EREBP)/Zea mays/AthaMap(0.585) More Information | Similar Motifs Found	motif file (matri
11	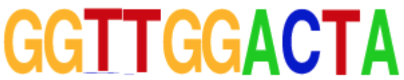	1e-17	-4.043e+01	0.14%	0.01%	48.9bp (69.7bp)	P(MYB)/Zea mays/AthaMap(0.651) More Information | Similar Motifs Found	motif file (matri
12	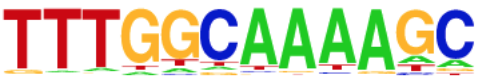	1e-17	-4.038e+01	2.10%	1.21%	55.6bp (55.3bp)	MA0020.1_Dof2/Jaspar(0.616) More Information | Similar Motifs Found	motif file (matri
13	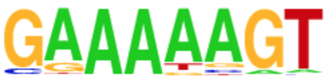	1e-17	-3.918e+01	21.04%	18.20%	56.3bp (57.1bp)	MA0021.1_Dof3/Jaspar(0.694) More Information | Similar Motifs Found	motif file (matri
14	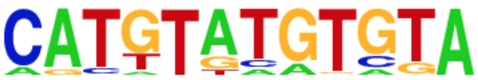	1e-16	-3.906e+01	0.38%	0.09%	63.0bp (74.7bp)	TEIL(AP2/EREBP)/Nicotiana tabacum/AthaMap(0.576) More Information | Similar Motifs Found	motif file (matri
15	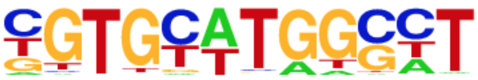	1e-16	-3.882e+01	0.10%	0.00%	53.3bp (0.0bp)	AtLEC2(ABI3/VP1)/Arabidopsis thaliana/AthaMap(0.679) More Information | Similar Motifs Found	motif file (matri
								motif

16	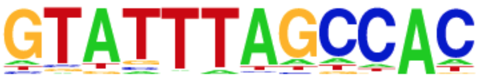	1e-16	-3.771e+01	1.07%	0.50%	53.2bp (57.0bp)	MF0005.1_Forkhead_class/Jaspar(0.536) More Information | Similar Motifs Found	file (matri
17	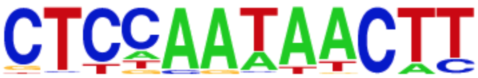	1e-16	-3.771e+01	0.31%	0.06%	49.9bp (47.3bp)	ATHB1(HD-ZIP)/Arabidopsis thaliana/AthaMap(0.567) More Information | Similar Motifs Found	motif file (matri
18	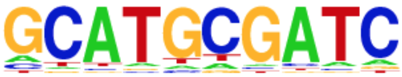	1e-15	-3.627e+01	3.82%	2.63%	54.2bp (57.0bp)	MA0565.1_FUS3/Jaspar(0.779) More Information | Similar Motifs Found	motif file (matri
19	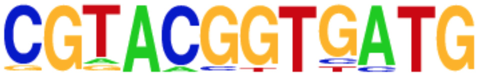	1e-15	-3.523e+01	0.09%	0.00%	54.3bp (0.0bp)	SPL14(SBP)/Arabidopsis thaliana/AthaMap(0.636) More Information | Similar Motifs Found	motif file (matri
20	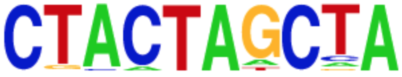	1e-14	-3.442e+01	0.53%	0.18%	57.3bp (55.6bp)	MYB.PH3(2)(MYB)/Petunia hybrida/AthaMap(0.529) More Information | Similar Motifs Found	motif file (matri
21	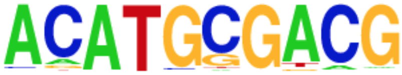	1e-14	-3.325e+01	0.38%	0.11%	59.5bp (62.1bp)	MA0565.1_FUS3/Jaspar(0.624) More Information | Similar Motifs Found	motif file (matri
22	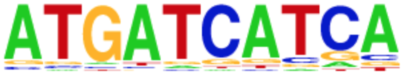	1e-14	-3.275e+01	9.53%	7.72%	55.9bp (58.3bp)	ZmHOX2a(2)(HD-HOX)/Zea mays/AthaMap(0.637) More Information | Similar Motifs Found	motif file (matri
23	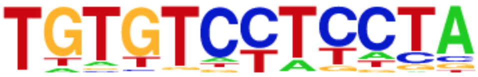	1e-14	-3.244e+01	0.11%	0.01%	52.2bp (33.6bp)	ID1(C2H2(Zn))/Zea mays/AthaMap(0.619) More Information | Similar Motifs Found	motif file (matri
24	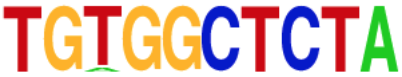	1e-13	-3.172e+01	0.09%	0.00%	58.1bp (3.1bp)	RAV1(1)(AP2/EREBP)/Arabidopsis thaliana/AthaMap(0.605) More Information | Similar Motifs Found	motif file (matri
25	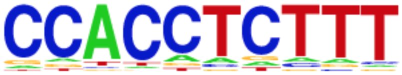	1e-13	-3.154e+01	7.42%	5.86%	55.5bp (58.6bp)	MA0128.1_EmBP-1/Jaspar(0.672) More Information | Similar Motifs Found	motif file (matri
26	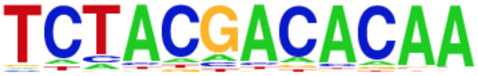	1e-13	-3.040e+01	2.10%	1.32%	53.8bp (59.3bp)	RAV1(1)(AP2/EREBP)/Arabidopsis thaliana/AthaMap(0.558) More Information | Similar Motifs Found	motif file (matri
27	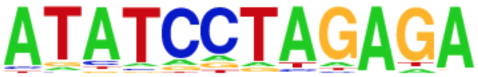	1e-12	-2.927e+01	2.60%	1.74%	52.5bp (55.5bp)	SD0003.1_at_AC_acceptor/Jaspar(0.641) More Information | Similar Motifs Found	motif file (matri
28	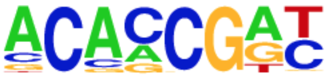	1e-12	-2.878e+01	2.10%	1.34%	56.8bp (61.6bp)	ABI4(2)(AP2/EREBP)/Zea mays/AthaMap(0.721) More Information | Similar Motifs Found	motif file (matri
29	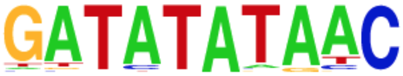	1e-12	-2.841e+01	2.58%	1.73%	55.5bp (60.0bp)	TBP(- other)/several species/AthaMap(0.668) More Information | Similar Motifs Found	motif file (matri
30	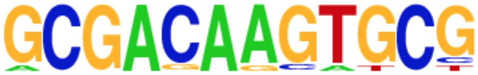	1e-12	-2.828e+01	0.08%	0.00%	45.5bp (0.0bp)	ARF1(ABI3/VP1)/Arabidopsis thaliana/AthaMap(0.634) More Information | Similar Motifs Found	motif file (matri
31 *	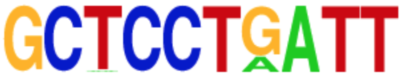	1e-11	-2.700e+01	0.15%	0.02%	52.7bp (52.4bp)	ZmHOX2a(1)(HD-HOX)/Zea mays/AthaMap(0.657) More Information | Similar Motifs Found	motif file (matri
32 *	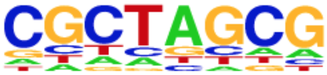	1e-10	-2.442e+01	29.60%	27.10%	55.5bp (60.7bp)	MA0565.1_FUS3/Jaspar(0.577) More Information | Similar Motifs Found	motif file (matri
33 *	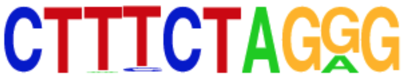	1e-9	-2.276e+01	0.20%	0.05%	58.7bp (49.9bp)	MA0064.1_PBF/Jaspar(0.617) More Information | Similar Motifs Found	motif file (matri

34 *	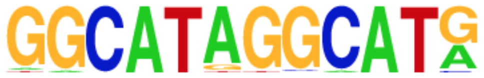	1e-9	-2.168e+01	0.06%	0.01%	49.3bp (63.7bp)	MA0581.1_LEC2/Jaspar(0.618) More Information | Similar Motifs Found	motif file (matri
35 *	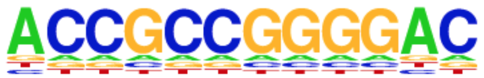	1e-9	-2.167e+01	0.09%	0.01%	40.6bp (32.6bp)	ABI4(1)(AP2/EREBP)/Zea mays/AthaMap(0.591) More Information | Similar Motifs Found	motif file (matri
36 *	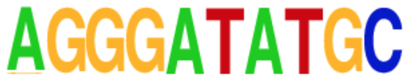	1e-9	-2.101e+01	0.08%	0.01%	42.6bp (28.0bp)	TaMYB80(MYB)/Triticum aestivum/AthaMap(0.759) More Information | Similar Motifs Found	motif file (matri
37 *	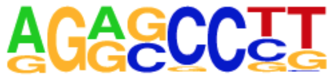	1e-8	-2.045e+01	2.80%	2.05%	55.0bp (55.8bp)	PCF2(TCP)/Oryza sativa/AthaMap(0.590) More Information | Similar Motifs Found	motif file (matri
38 *	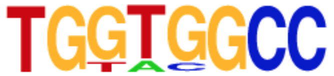	1e-8	-2.004e+01	1.45%	0.93%	55.2bp (60.9bp)	MA0567.1_ERF1/Jaspar(0.655) More Information | Similar Motifs Found	motif file (matri
39 *	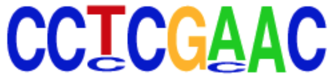	1e-7	-1.809e+01	0.60%	0.31%	55.7bp (59.7bp)	MA0553.1_SMZ/Jaspar(0.825) More Information | Similar Motifs Found	motif file (matri
40 *	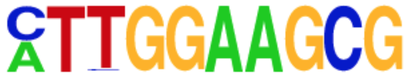	1e-7	-1.615e+01	0.09%	0.01%	49.0bp (32.0bp)	POL008.1_DCE_S_I/Jaspar(0.710) More Information | Similar Motifs Found	motif file (matri
41 *	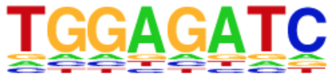	1e-6	-1.572e+01	0.96%	0.60%	58.6bp (58.3bp)	ZmHOX2a(2)(HD-HOX)/Zea mays/AthaMap(0.715) More Information | Similar Motifs Found	motif file (matri
42 *	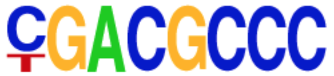	1e-6	-1.482e+01	0.17%	0.05%	47.0bp (61.7bp)	NtERF2(AP2/EREBP)/Nicotiana tabacum/AthaMap(0.746) More Information | Similar Motifs Found	motif file (matri
43 *	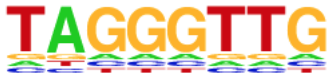	1e-5	-1.372e+01	0.68%	0.40%	54.4bp (56.1bp)	P(MYB)/Zea mays/AthaMap(0.662) More Information | Similar Motifs Found	motif file (matri
44 *	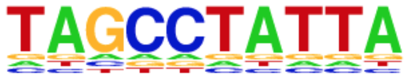	1e-5	-1.212e+01	0.07%	0.01%	48.3bp (40.3bp)	POL004.1_CCAAT-box/Jaspar(0.606) More Information | Similar Motifs Found	motif file (matri
45 *	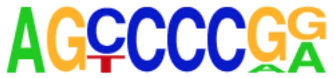	1e-5	-1.210e+01	0.69%	0.42%	52.8bp (56.6bp)	ABI4(2)(AP2/EREBP)/Zea mays/AthaMap(0.648) More Information | Similar Motifs Found	motif file (matri
